# Knowledge and awareness level of health undergraduate students on child abuse: a cross-sectional study

**DOI:** 10.1590/1806-9282.20231742

**Published:** 2024-07-19

**Authors:** Alev Üstündağ, Ayşe Göktaş, Çiğdem Aytekin

**Affiliations:** 1 University of Health Sciences, Gülhane Faculty of Health Sciences, Department of Child Development – Ankara, Turkey.; 2 University of Health Sciences, Gülhane Faculty of Health Sciences, Department of Occupational Therapy – Ankara, Turkey.; 3 Hacettepe University, Faculty of Health Sciences, Department of Child Development – Ankara, Turkey.

**Keywords:** Child abuse, Child neglect, Students, public health

## Abstract

**OBJECTIVE:**

The aim of the study was to investigate the level of knowledge of symptoms and risks of child abuse among undergraduate health science students according to their socio-demographic characteristics.

**METHODS:**

This is a cross-sectional study involving 485 student volunteers. The data collection tools used in the study were the Demographic Data Collection Form and the Scale for Diagnosing Symptoms and Risks of Child Abuse and Neglect.

**RESULTS:**

It was found that health students' knowledge of the symptoms and risks of child abuse was moderate. It was also found that knowledge of diagnosing the symptoms and risks of child abuse was higher among women than among men, higher among those who had received education on child abuse, and increased with grade level.

**CONCLUSION:**

Child abuse is an important public health issue, and there is a need to raise awareness of this issue among health students.

## INTRODUCTION

Child abuse, which is a significant social and health threat today, is defined by the World Health Organization (WHO)^1^ as "*any physical and/or emotional maltreatment, sexual abuse, neglect and commercial exploitation of children under the age of 18 that results in actual or potential harm to the health, development or dignity of the child*." According to the WHO report, 23% of children were subjected to physical abuse, 16% were subjected to physical neglect, 36% were subjected to emotional abuse, and 18% of girls and 8% of boys were subjected to sexual abuse^1^. In Turkey, although the studies on child abuse are limited, it can be observed that the rates are at a considerable level^2^.

Child abuse is associated with a wide range of long-term negative health and developmental outcomes, extending into adolescence and young adulthood^3^. Early identification of child abuse is therefore important. Health professionals who encounter children and are likely to encounter children have an important role to play in the early identification of child abuse^4^. Health professionals have responsibilities not only in the area of child health but also in the area of child protection^5^. The most important of these responsibilities is the early recognition of symptoms of abuse or endangerment in terms of child welfare^5^. For this reason, all health professionals, regardless of their field of work, need to recognize the early signs and risks of child abuse^6^. In the hospital environment, healthcare professionals frequently encounter abused children. However, a lack of awareness of the early signs and risks of child abuse and inadequate knowledge on this topic may lead to cases that come to the hospital environment being missed^7^.

Given that all children are at risk of child abuse, it is important and necessary for undergraduate health students to have knowledge about diagnosing the symptoms and risks of child maltreatment, especially as future health professionals. Although the studies on the level of knowledge about child abuse among students studying in undergraduate programs related to health sciences are quite limited, it has been found that students' knowledge about this topic is insufficient, their level of identification of symptoms and risks is not at the desired level, and they need information about this topic^8,9^. In addition, according to the results of the research, public students know very less about sexual infection disease and male adolescents are less interested in health services^10^. Based on these findings, our study aimed to investigate the level of knowledge of the symptoms and risks of child maltreatment among undergraduate health science students according to their socio-demographic characteristics.

## METHODS

### Research model

This is a cross-sectional study.

### Participants

In this research, the study group comprised 485 volunteer students at Ankara, who are continuing their undergraduate programs related to health sciences. The sample size was determined with the G*POWER 3.1.9.4 statistics software used in cases where the population was known. Of the 485 students included in the study, 88% (n=427) were female, 12% (n=58) were male, and the mean age was 21.46±2.22 years.

### Measurement

The data regarding the research were collected using the Demographic Data Collection Form and the Scale for Diagnosing Symptoms and Risks of Child Abuse and Neglect forms through the face-to-face interview method.

#### Demographic Data Collection Form

The information form was prepared by the researchers. It consists of 11 questions, including the socio-demographic characteristics of the students.

#### Scale for Diagnosing Symptoms and Risks of Child Abuse and Neglect

The scale developed by Uysal^11^ was designed to assess the competence of health professionals in recognizing the symptoms and risks of child abuse and neglect and in differentiating between the risks. The 5-point Likert scale consists of 67 questions. The scale has six sub-dimensions: Physical Symptoms of Child Abuse (PSCA), Behavioral Symptoms of Child Abuse And Neglect (BSCAN), Symptoms of Child Neglect (SCN), Characteristics of Parents Prone to Abuse and Neglect (CPPAN), Characteristics of Children Prone To Abuse and Neglect (CCPAN), and Family Characteristics in Child Abuse and Neglect (FCCAN). In this study, the Cronbach's alpha value of the scale was found to be 0.83.

### Data analysis

Mean and standard deviation were used as descriptive statistics for continuous data. The Kolmogorov-Smirnov test was used to test for normality, and the Student's t-test in independent groups was used to assess the distribution of continuous variables that fit the normal distribution in two groups, and one-way ANOVA was used to assess the distribution in three or more groups. The distribution of non-normally distributed variables in two groups was compared by the Mann-Whitney U test, and the distribution in three or more groups was compared by the Kruskal-Wallis test. The Dunn-Bonferroni test was used as a multiple comparison test for the Kruskal-Wallis test to find the source of the difference.

The relationship between the variables was assessed by point bi-serial and Spearman correlation analysis. The cutoff points used for the interpretation of the correlation coefficients obtained are as follows: 0.00–0.19 very weak, 0.20–0.39 weak, 0.40–0.69 moderate, 0.70–0.89 high, and 0.90–1.00 very high. The Statistical Package for the Social Sciences Statistics for Macintosh (version 21.0; IBM Corp., Armonk, NY, USA) was used for all statistical analyses performed within the scope of the study. The statistical significance level was set at 0.05.

Ethics Committee approval of the study was obtained with the meeting decision of the University of Health Sciences, Gülhane Scientific Research Ethics Committee (dated 05.12.2023 and No. 2023/359).

## RESULTS

According to the results of the research, the mean total score of the female participants was 3.61±0.30 and the mean total score of the male participants was 3.51±0.35. Accordingly, the students' level of diagnosing the symptoms and risks of child neglect and abuse is at a moderate level. Table 1 shows the results of the comparison of the scores of students according to some demographic information.

**Table 1:** Comparison of total scale scores and sub-dimension total scores according to some demographic information.

		PSCA (Mean±SS)	BSCAN (Mean±SS)	SCN (Mean± SS)	CPPAN (Mean±SS)	CCPAN (Mean ±SS)	FCCAN (Mean ±SS)	SDSRCAN (Mean±SS)
Gender	Women n=427	3.85±0.40	3.94±0.57	3.73±0.39	3.61±0.48	3.26±0.42	2.79±0.33	3.61±0.30
	Men n=58	3.67±0.51	3.80±0.76	3.59±0.49	3.65±0.44	3.19±0.47	2.76±0.46	3.51±0.35
Test statistics**		z=-2.573 p=0.010	z=-1.199 p=0.231	z=-2.270 p=0.023	z=0.542 p=0.588	z=-1.526 p=0.127	z=0.002 p=0.999	z=-2.178 p=0.029
Grade level	1 n=122	3.75±0.40	3.86±0.60	3.62±0.40	3.47±0.43	3.20±0.39	2.81±0.36	3.52±0.27
	2 n=107	3.81±0.40	3.86±0.60	3.63±0.40	3.50±0.46	3.20±0.42	2.85±0.35	3.56±0.31
	3 n=97	3.81±0.42	3.95±0.60	3.73±0.35	3.68±0.43	3.31±0.38	2.76±0.36	3.62±0.29
	4 n=146	3.93±0.43	4.00±0.59	3.80±0.45	3.78±0.51	3.30±0.47	2.75±0.34	3.68±0.33
Test statistics*		**χ** ^2^=11.077 p=0.011 4>1	**χ** ^2^=3.410 p=0.333	**χ** ^2^=9.502 p=0.023 4>1	**χ** ^2^=33.879 p<0.001 3>1,2 4>1,2	**χ** ^2^=8.527 p=0.036	**χ** ^2^=2.607 p=0.456	**χ** ^2^=17.048 p=0.001 4>1
Relatives living at home during childhood	Yes n=103	3.88±0.40	4.00±0.52	3.77±0.42	3.70±0.48	3.30±0.41	2.72±0.33	3.64±0.29
	No n=382	3.81±0.42	3.90±0.62	3.69±0.41	3.60±0.47	3.24±0.43	2.81±0.35	3.59±0.31
Test statistics**		z=-1.283 p=0.200	z=-1.483 p=0.138	z=-0.922 p=0.357	z=-1.661 p=0.097	z=-1.500 p=0.134	Z=2.354 p=0.019	t=1.689 p=0.092
Status of education about child abuse	Yes n=136	3.87±0.39	3.98±0.56	3.74±0.40	3.72±0.49	3.25±0.43	2.83±0.38	3.65±0.29
	No n=349	3.81±0.43	3.90±0.61	3.70±0.41	3.57±0.46	3.25±0.42	2.78±0.34	3.58±0.31
Test statistics**		z=-1.237 p=0.216	z=-1.332 p=0.183	z=-1.004 p=0.316	z=-2.830 p=0.005	z=-0.097 p=0.922	z=-0.699 p=0.484	t=2.196 p=0.029
Status of believing that knowledge about child abuse is adequate	Yes n=107	3.91±0.46	3.98±0.63	3.80±0.45	3.67±0.52	3.27±0.50	2.86±0.37	3.67±0.33
	No n=378	3.80±0.40	3.91±0.59	3.69±0.39	3.60±0.46	3.24±0.40	2.77±0.34	3.58±0.30
Test statistics**		z=-2.202 p=0.028	z=-0.820 p=0.412	z=-1.875 p=0.061	z=-0.896 p=0.370	z=-0.611 p=0.541	z=-1.804 p=0.071	t=2.612 p=0.009

* Kruskal-Wallis test statistics.

** Mann-Whitney U test statistical value.

Bold indicates p<0.05.

The results of the comparison of the scores of students on the SDSRCAN scale according to the departments they attend are presented in Table 2, and the results of the correlation analysis are presented in Table 3.

**Table 2:** Comparison of the total and sub-dimension total scores of the Scale for Diagnosing Symptoms and Risks of Child Abuse and Neglect scale according to the departments attended by the students.

		PSCA (Mean±SS)	BSCAN (Mean±SS)	SCN (Mean±SS)	CPPAN (Mean±SS)	CCPAN (Mean±SS)	FCCAN (Mean±SS)	SDSRCAN(Mean±SS)
Programs	Nutrition and Dietetics^1^ n=34	3.96±0.36	3.93±0.56	3.76±0.35	3.66±0.43	3.34±0.41	2.86±0.25	3.67±0.25
	Child Development^2^ n=127	3.76±0.38	3.87±0.59	3.69±0.40	3.71±0.47	3.21±0.41	2.79±0.36	3.58±0.28
	Speech and Language Therapy^3^ n=17	3.73±0.36	3.83±0.52	3.61±0.28	3.63±0.36	3.27±0.31	2.76±0.28	3.54±0.27
	Dental Medicine^4^ n=16	3.45±0.37	3.55±0.52	3.49±0.37	3.36±0.47	3.04±0.51	2.83±0.35	3.35±0.25
	Midwifery^5^ (n=15)	3.87±0.45	3.86±0.60	3.59±0.38	3.60±0.51	3.37±0.48	2.81±0.44	3.59±0.27
	Pharmaceutics^6^ n=25	3.86±0.40	3.90±0.66	3.73±0.40	3.53±0.41	3.37±0.39	2.76±0.35	3.60±0.29
	Ergotherapy^7^ n=31	3.78±0.28	3.97±0.56	3.74±0.30	3.64±0.45	3.32±0.43	2.67±0.39	3.59±0.24
	Physiotherapy and Rehabilitation^8^ n=28	3.98±0.39	4.12±0.68	3.95±0.45	3.76±0.48	3.26±0.43	2.60±0.37	3.72±0.35
	Nursing^9^ n=81	3.94±0.45	4.09±0.61	3.80±0.44	3.63±0.51	3.29±0.41	2.87±0.30	3.68±0.34
	Audiology^10^ n=21	3.65±0.40	3.63±0.45	3.38±0.39	3.40±0.46	2.93±0.41	2.98±0.43	3.40±0.26
	Health Administratio^11^ n=22	3.72±0.34	3.95±0.53	3.63±0.34	3.39±0.53	3.14±0.37	2.77±0.29	3.50±0.30
	Social Services^12^ n=33	3.83±0.6	4.05±0.63	3.65±0.36	3.45±0.45	3.23±0.36	2.79±0.40	3.56±0.29
	Medicine^13^ n=35	3.97±0.52	3.87±0.59	3.85±0.49	3.66±0.43	3.38±0.48	2.74±0.27	3.68±0.37
Test statistics*		**χ** ^2^=41.073 p<0.001	**χ** ^2^=27.115 p=0.007	**χ** ^2^=39.140 p<0.001	**χ** ^2^=24.650 p=0.017	**χ** ^2^=26.687 p=0.009	**χ** ^2^=24.428 p=0.018	**χ** ^2^=37.383 p<0.001
Source of difference**		4<1 4<8 4<9 4<13	9>4 9>10	10<1 10<8 10<9 10<13		10<6 10<9 10)<13	8<9 8<10	4<1 4<8 4<9 4<13 10<8 1<9

* Kruskal-Wallis test statistics.

** Dunn-Bonferroni post hoc test results.

Bold indicates p<0.05.

**Table 3:** Analyses of the relationship between some variables and scale total and subscale.

		PSCA	BSCAN	SCN	CPPAN	CCPAN	FCCAN	SDSRCAN
Gender	r_pb_	-0.131	-0.078	-0.109	0.027	-0.054	-0.028	-0.101
	p	0.004	0.088	0.016	0.558	0.238	0.538	0.026
Status of education about child abuse	r_pb_	-0.068	-0.063	-0.048	-0.137	0.002	-0.065	-0.099
	p	0.135	0.169	0.294	0.002	0.969	0.155	0.029
Status of believing that knowledge about child abuse is adequate	r_pb_	-0.103	-0.045	-0.119	-0.058	-0.027	-0.107	-0.118
	p	0.023	0.319	0.009	0.203	0.554	0.019	0.009
Grade level	rho	0.160	0.096	0.170	0.269	0.114	-0.084	0.208
	p	0.000	0.036	0.000	0.000	0.013	0.069	0.000
Programs	r_pb_	0.103	0.092	0.074	-0.098	0.016	-0.014	0.053
	p	0.025	0.046	0.107	0.033	0.724	0.756	0.249

r_pb_: Point bi-serial correlation. rho: Spearman correlation.

Bold indicates p<0.05.

## DISCUSSION

When the results of the study were analyzed, it was found that the total score of the SDSRCAN scale and the subscale scores of the PSCA and SCN differed significantly according to gender and that the mean scores of women were higher. In other words, women are better at diagnosing risk symptoms of child abuse and neglect, physical symptoms of abuse in the child, and behavioral symptoms in the child. Similarly, in the study conducted by Güdek-Seferoğlu et al.^12^, they investigated the level of SDSRCAN of nursing students, and in the study conducted by Pesen and Epçaçan^13^ with pre-service teachers, the mean score of women was found to be higher than that of men. According to the results of the study conducted by Türk et al.^14^ which aimed to investigate the level of SDSRCAN among university students, the total mean score of the scale and the mean scores of the subscales of PSCA, BSCAN, SCN, and FCCAN are higher among females. In the studies of Tek and Karakaş^15^ and Ozbey et al.^16^ which investigated the level of knowledge and awareness of child neglect and abuse among nursing students, and in the study of Jeong et al.^17^ which investigated the awareness and intention to report child neglect and abuse among nursing and education students, the mean scores of female students were higher than male students.

According to the results obtained, it was found that the grade level of the students made a significant difference in the level of the SDSRCAN. Accordingly, the total score of the SDSRCAN and the subscale scores of the PSCA, SCN, CPPAN, and CCPAN increased as the grade level increased and the score of the fourth-grade students was significantly higher than that of the first-grade students. In line with the findings of this study, Poreddi et al.^18^ examined the level of knowledge of nursing students on child abuse and neglect and found that the students' knowledge and attitudes toward child abuse increased as they progressed through the academic year.

Another finding from the study was that the mean scores of students who had received training on child abuse, and those who felt their knowledge was sufficient, were significantly higher. Looking at the literature, some studies conclude that the level of awareness is higher among those who receive child abuse training^15,19,20^. The results of the research and the literature are consistent. Increasing the knowledge of individuals is important not only for their future health but also for preventing the negative consequences of violence and abuse^21^.

## CONCLUSION

The study found that undergraduate health students' knowledge of recognizing the symptoms and risks of child abuse and neglect was average. In this context, our study makes a general contribution to the literature on international health education. Therefore, in an international context, it is recommended that students should be given more detailed information on this subject in the undergraduate curriculum and that scientific activities should be carried out to increase students' awareness.

In addition, the strengths of this study are as follows. The findings were obtained through adolescents' self-reports, and it has a rigorous approach to data analysis. Although this study produced important results, it is important to mention its limitations. Our relatively small sample and one-off design limit the broad applicability of our findings. Therefore, further studies are needed to make the findings more generalizable. Future research should be conducted at regular intervals in larger sample groups. However, it should be noted that this study is only a preliminary investigation of a topic that requires further research. Furthermore, as this is a cross-sectional study, it is not possible to make a causal inference from our results. A potential limitation of the research is that it relied on self-reported data. Self-reported measures may not always accurately reflect actual behavior or awareness levels of participants. Hence, future studies may consider including observational or behavioral measures to supplement self-report data.
